# "The Hospital" Nursing Mirror

**Published:** 1899-12-23

**Authors:** 


### "The Hospital" Nursing Mirror.

Being the Nursing Section of "The Hospital."

[Contributions for this Section of "The Hospital" should be addressed to the Editor, The Hospital, 28 & 29, Southampton Street, Strand, London, W.C., and should have the word "Nursing" plainly written in left-hand top corner of the envelope.]

### Notes on News from the Nursing World.

### A NURSE'S EXPERIENCES ON AN AMBULANCE TRAIN IN SOUTH AFRICA.

A correspondent from Durban writes:—

"I have had the pleasure of being a passenger for a short distance on board an ambulance or hospital train. The sergeant in charge was the late dispenser at the Station Hospital, Pietermaritzburg. Tall, strong, well built, of a most amiable and genial disposition, he is quite the right man in the right place, and should be a great comfort and source of confidence to the wounded under his charge. The beds are very comfortable in appearance, and all the appointments are specially adapted to the necessities and emergencies of the journey by rail. Two of the carriages are arranged in Russian style—the bed stretchers running parallel with the line. The wounded borne on these are not under the necessity of being removed to another bed on arrival at the train; the stretcher is just slung on its support, and at the completion of the journey the patient is easily removed with less risk of fatigue and injury to wounds. The wounded are under a most competent medical officer, and are very appreciative of all that is being done for their comfort as they travel by road or rail. I also went to see Addington Hospital, which has a very good position quite close to the beach. From its verandah and balcony a beautiful view is obtained of both the bay and bluff, at this time more interesting to convalescent patients, as so many ships are at the outer anchorage. The building itself is in the form of a square, with a quadrangle in the centre, is admirably ventilated, and kept in spotless purity. Flowers, artistically arranged, are in every ward. The hospital has a staff of matron and 22 nurses, all the beds being occupied. There is room for about 200 patients. The nurses' home, a recent acquisition, is a very great boon. Each nurse has her own bedroom, and on each landing there is lavatory and bathroom accommodation. The night nurses have their sleeping apartments upstairs. There are also large drawing and dining-rooms, which can be utilised as one room by a sliding door arrangement."

### NURSING AT LADYSMITH.

Another South African correspondent, who was assisting the nurses at Intombi, the neutral camp near Ladysmith, writes that the nurses told her they had such hard work and such poor food, yet, strange to say, their health in no way suffered. This was probably due to the fact that they were so much in the open air, continually going from tent to tent, carrying food from the hot little wood and iron kitchen, where our correspondent, with the help of her own native servant, did the cooking for a week, with a scanty allowance of sleep. During the whole time she confesses that she was "seldom cool and never clean." One of the Sisters from Netley, who had been in the Soudan, assured her that the wounds were far worse than those of any of the troops she had to nurse in Egypt. We also hear that sickness is becoming serious in Durban itself. Of course, the place is immensely over-crowded, and some of the poorest of the refugees easily fall victims to fever and kindred diseases. The hospitals are already turning out patients not fit to leave, because their beds are more urgently required for severer cases.

### "FINE LADIES AS NURSES."

A protest has been made against sending out to South Africa "fine ladies as nurses." We entirely concur in the opinion that persons who can only be described as "fine ladies" should not be dispatched to nurse the sick and wounded. But is there any evidence to justify the insinuation that this is being done? So far as the War Office is concerned none but fully trained nurses, most of them of ripe experience, have been engaged. That they will behave as ladies we are satisfied, but it may be assumed that there is no objection to ladies—only to ladies who have not been trained to nurse. The help of any of the latter who have gone, or are going, to the seat of the war may be useful; but they will not, we assume, be employed as nurses; neither will the expenses of their journey be defrayed out of any of the public subscriptions.

### AN UNKNOWN HERO.

Last Saturday week afternoon a fine stalwart-looking man was admitted to St. Mary's Hospital with an injury to his knee. Whilst waiting to be seen he began to talk to the casualty porter (himself an old soldier, who worked under General Buller when he was a colonel) about the war, and told him that he had come over from America the day before to join the Reserve, but that while he was on his way that morning to St. George's Barracks he slipped as he was getting into an omnibus and hurt his knee. One of the Nurses writes:—

"Hearing that we had a hero in our midst we went to see for ourselves. He was so quiet—pain never troubled him—he was like one looking at a fixed object far away, and wholly occupied with that alone. He said he had been all through the Spanish-American war under an assumed name, had seen several skirmishes and a few battles, had been wounded, and after a time paid off. Then he went to Texas, where he met several fellow-countrymen. He stayed for some time in San Antonia, where several horses have been bought to go to South Africa. He travelled along to Kansas, and while he was there he met Captain Marsham buying mules for the British Army. The first thing he saw in the papers when he got to New York was that England needed all her Reserves. He applied at once to the British Consul, who sent him back to England. 'Nurse,' said he, 'I am just longing to give them Boers a bit of my mind through the end of my bayonet; but here I am. Do you think I have broken my leg? I feel I am going to the war somehow.' His injury, on examination, turned out to be a stellate fracture of the patella. In a few days he was up on crutches, and has just left us—full of hope, certain of getting in time the desire of his heart. He spent much time alone, sitting by the fire evidently plotting a fresh plan of campaign. Almost his last words were, 'Nurse, I'm what people call a lucky chap, and, please God, we'll gain the victory yet; only I must get there somehow, I must.'"

### THE NURSING OF WOUNDED SOLDIERS AT CONVALESCENT HOMES.

There is no need more generally recognised than that of an ample supply of convalescent homes for the nursing of our soldiers when they are discharged from the military hospitals. In the face of this fact the failure of the War Office to respond to a very generous offer made by Mrs. Montagu Fowler is rather surprising. Some weeks ago Mrs. Fowler offered to provide for our soldiers and sailors, wounded and invalided in the war, a convalescent home situated on the river within easy access of Woolwich and the Herbert Hospital, with accommodation for 150 men, in the fine building she had selected for the purpose; to find the entire staff, including a matron, nurses, and orderlies, and to do this without any appeal to the public. She announced her willingness to take the sole responsibility of the management, backed by the knowledge that she had secured a promise of advisory help from one of the foremost and most experienced organisers of similar undertakings in London, as well as the services of a matron of 17 years' hospital experience. Although this offer was supplemented by an undertaking on the part of the trustees of Lloyd's Patriotic Fund to pay the expenses of as many convalescents as might be sent to the home, it has been allowed to lapse, and on Tuesday Mrs. Fowler was obliged to announce that, owing to her early departure for Egypt, where her husband is due in January as chaplain to the Bishop of Jerusalem, she could not renew it. We do not think it is fair to assume that the War Office authorities alone do not realise the extreme importance of providing sufficient convalescent homes; but some explanation of the apparent indifference to such a timely and gracious proposal as that of Mrs. Fowler ought to be forthcoming.

### THE WAR AND INDEPENDENT NURSING FUNDS.

AN army chaplain having informed the *Church Times* that he has given a collection to assist the fund which the Kilburn Sisters have started for nursing the sick and wounded, our contemporary makes the following comments: "There is not the least indication in the appeal which has been sent out broadcast that the Sisters' services would be accepted by the local authorities, nor is the Archbishop of Cape Town's name mentioned. It is one thing to offer personal service in the nursing of the sick and wounded; it is quite another to be starting an independent fund in rivalry to the many that are now before the public." This is exactly the stand we have taken from the first in the matter, and we are glad to find that our attitude is supported by the leading High Church journal.

### THE WALTHAM TRAINING SCHOOL FOR NURSES.

THERE are many interesting points in the report of the Waltham Training School for Nurses for the year ending August 31st, 1899, which has just reached us. We notice now only the reference made to the attention given by the managers to instruction in the art of housekeeping. The principle at the foot of the instruction is that nursing is "essentially home-making for the sick, and that only those proficient in home-making for the well can ever become first-class nurses." Miss Boland, the lady in charge of the department, devotes eight hours a week for four months of each term to this subject. She gives a course of lectures and demonstrations in the special chemistry of domestic science. Laboratory work is also required of the student nurses, who have to prove their understanding of the questions by performing all the tests and experiments employed in the course. The course in dietetics consists of twelve lectures, and there are sixteen exercises in family cooking and cooking for the sick, where the actual work of preparing and serving the food is carried on. This is how the instruction given is carried out in practice:—

"Three probationers serve in the kitchen. A new one goes on duty every tenth day. She first prepares and cooks the vegetables, soups, and meats, is responsible for the fire, cleanliness of utensils, sink, and kitchen. At the end of ten days she is promoted to making the breads, sauces, porridge, tea and coffee, and keeping tidy the pantries. In the last ten days she has an oversight of the whole, writes the orders, takes care of the supplies, makes the cakes and puddings, and helps in teaching her successors. While three probationers are thus employed, others have the care of the chambers, and are taught bed-making, sweeping, dusting, and cleansing of vessels. By turns two wait on table, care for the china and silver, and keep in order the dining-room. Another probationer is responsible for the lamps, nurses' parlour, and office. The probationers serve one month in each of these branches of housekeeping, and only those who show aptitude for each work are retained in the school."

### THE NURSES' HOME AT CATERHAM ASYLUM.

A MOST enjoyable evening was spent at this asylum on Monday at the new nurses' home. A picture of the medical superintendent (Dr. G. Stanley-Elliot) was presented by the members of the female staff to the home, as a token of their sincere regard and appreciation of his untiring energy, both in the erection and furnishing of what is now acknowledged by all to be a most comfortable and luxurious building. After the presentation, tea and coffee was handed round, followed by music, recitations, and other amusements.

### OUR CLOTHING DISTRIBUTION.

WE have another list of welcome contributions to acknowledge this week: From the Editor of THE HOSPITAL, £1 1s.; Miss Pritchard, 5s.; Mr. Wilcox, 2s. 6d.; and Miss Good, 2s. 6d. This money was spent to great advantage at Messrs. Garrould's, in Edgware Road, who allowed a most liberal discount. Nurse Sykes sent a small packet of cards, 10 toys, and 14 garments; Nurse Coverdale, three garments; Miss M. A. Stout, 13; Nurse Lucy, 13; J. W., five; Miss Henslowe, a nice parcel; Nurse Cunningham, a contribution of boxes, &c., with a tobacco-purse (stocked); Miss T. E. S., a scarf; No. 37, a suitable selection; Nurse Knight, a goodly package of children's garments made from Viyella flannel; and Policy No. 544, 37 garments and useful gifts. Space does not permit us to do justice to the number and usefulness of the garments sent, nor yet to the admirable workmanship that has transformed wool and flannel into dainty clothing. Our contributors have purchased their materials in no niggard spirit, and their generosity cannot fail to be appreciated. It would be a great pleasure to describe them fully, but there are too many to mention individually, and one deserves as great praise as another. Next week we shall be able to give full particulars about the distribution.

### NURSES WHO ARE TRAINED BY NURSING ASSOCIATIONS.

UNDER the auspices of the Northumberland Nursing Association a branch association for Newbiggin has been formed. Miss White, the superintendent of the parent association, attended the inaugural meeting, and alluded to its progress. Started two years ago, with five nurses, it has since been the means of scattering over the country twenty-eight. Having explained that the association supplies two classes of nurses—fully-trained hospital nurses, who worked principally in largely populated places and paid daily visits; and village nurses, who were mostly daughters of working men, and whom they trained themselves—Miss White said that the former required a salary of £80, and the latter one of £50 per year. Asked whether the nurses which they trained themselves could attend various operations, Miss White answered in the affirmative, and added that "they worked entirely under the doctors." All the same, we sympathise with the lady who expressed a wish to have "the very best class of nurse," which we presume the other nurses do also. The Newbiggin Association do not seem to have yet decided whether they will pay £50 or £80; but the parish is rich enough to afford to employ a fully-trained nurse. The nurses who are trained by the association are better than none at all, but they must obviously be of a very inferior type.

### MILITARY HONOURS FOR HOSPITAL NURSES.

It has been the distinction of the Colchester people to give a military funeral to a hospital nurse. Sister Barbara Milne, the superintendent of the Station Garrison Hospital, whose mortal remains were thus honoured, only lately joined the Eastern District, succeeding a nurse who has gone to South Africa. Except that there was no firing party at the grave, the funeral was similar to that accorded to the British soldier. The funeral procession was headed by the band of the Royal Warwickshire Regiment, who played Mendelssohn's Funeral March *en route*, and the coffin, shrouded in the Union Jack, was drawn on a gun, behind which followed several officers and the 9th Bearer Company of the Royal Army Medical Corps.

### BOARD MONEY OR RATIONS?

At a recent meeting of the Caistor Board of Guardians, the proposal was put forward by the Visiting Committee that the assistant nurse should have 7s. a week in lieu of rations. The nurse had pointed out that while the rations supplied were often more than she needed, she could sometimes eat something different to what was allowed, and she thought that it would be more satisfactory to both parties if she were allowed 7s. in place of rations. Mr. Stevens, one of the Local Government Board inspectors, happened to be sitting with the Board, and the Chairman asked his views on the matter. Mr. Stevens said he did not know whether the Local Government Board would accede or not. The question, he continued, was not unattended by difficulties. "For instance, a nurse in an urban district who was placed on this system, was found to be using for herself the milk intended for children; and there were other cases where officers had been tempted to take from the rations of patients for themselves, and thus pocketed the whole of the money allowed them." It strikes us, however, as ridiculous to presuppose that because one nurse was tempted to dishonesty, every nurse placed in the same position must necessarily follow her example. We think that the true solution of this question, which is by no means confined to Caistor, lies in the substitution of "board" for "rations."

### LITTLE CHILDREN, LOVE ONE ANOTHER.

Few people realise the excellent work that is being done by the Children's Happy Evenings Association. Every winter the lives of thousands of little Board School children in every part of London are brightened by its efforts. Dolls and toys, paint-boxes and games, soon bring in their train the sound of merry laughter and the glisten of bright eyes. In the midst of their own pleasure and gladness the children do not forget their less fortunate brothers and sisters. In one of the Hoxton schools some of the little girls have spent their happy evenings for many weeks in dressing dolls for the sick children of their nearest hospital. On Saturday last a very happy little band arrived at the Metropolitan Hospital bearing the results of their labours. There were about a dozen dolls in all, dressed in every variety of colour and material. Soon the little invalids were rejoicing in their new-found treasures, hugging their dollies and gazing in awe and wonder at the dainty gowns and bonnets. This was a new Santa Claus! The little donors, to whom a doll "of their very own" is an almost unknown quantity, went home in a glow of blissful joy. They felt amply rewarded for all their work, and their only anxiety was to know how soon they might begin the dressing of a fresh batch of dolls for "the poor little children in the hospital."

### ST. MARY'S HOSPITAL.

The sale of work at St. Mary's Hospital was a great success—£70 was made. The Board-room looked quite pretty, surrounded by stalls laden with bright coloured wares, fancy work, pictures, &c. The matron and several sisters and nurses were the saleswomen, and right well they did their work. The refreshment table was a special attraction—a cup of tea and as many cakes as you could eat for sixpence. As the day wore on and the hour of closing drew near the prices were reduced, especially to the nurses of the hospital.

### THE SALARIES OF NURSES AT PEMBURY WORKHOUSE.

There has been a discussion at a meeting of the Tonbridge Board of Guardians on the simultaneous resignation of three nurses. It transpired that one was leaving to be married, another for a better appointment, and the third because the superintendent was leaving. In the first case there was obviously a very sufficient reason, but it seems that in the second the nurse was exchanging Pembury for Kingston Workhouse because the Guardians of the latter Union pay a salary of £30 a year as against £26 paid by the former. It is not unusual for nurses to change their appointments for better ones, and we agree with those of the Tonbridge Guardians who urged that the revision of the salaries would help to stop the resignations which have lately been so frequent. It is, however, a little unusual for a nurse to throw up a post simply on the ground that she objects to work under a new superintendent.

### SHORT ITEMS.

There will be nursing the wounded at the Seamen's Branch Hospital in the Royal Victoria and Albert Docks. Lord Lansdowne has arranged with the Seamen's Hospital Society that a number of beds shall be placed at the disposal of the War Office for the reception of the soldiers who, on their arrival in the Albert Docks from South Africa, may not be fit to at once travel further.—The dearth of nurses willing to serve under Boards of Guardians was illustrated at a meeting of the Newton Board the other day, when it was announced that there was only one application for the position of nurse. Yet Newton Abbot is a pleasant Devonshire town, close to delightful watering-places, and we are not aware that the Guardians have made themselves odious of late.—The matron of St. Bartholomew's Hospital writes us that Baroness Emily Halkett, who has gone out to South Africa with the view of rendering any assistance she can in nursing, was in the service of that hospital as probationer for 18 months about 10 years ago. Miss Stewart adds, "She passed no examination, and holds no certificate of efficiency from here. She, therefore, cannot be said to be trained here."

### Lectures to Surgical Nurses.

By H. A. LATIMER, M.D. (Dunelm), M.R.C.S. of Eng., L.S.A. of London, Consulting Surgeon, Swansea Hospital; Past President of the Swansea Medical Society; Lecturer and Examiner of the St. John Ambulance Association, &c.

MALIGNANT DISEASES (*continued*).

MALIGNANT diseases, about which I was lecturing last week, vary very much in their rate of progress, and even in their fatality, with the variety which has affected the sufferers. Starting with that form called epithelioma, as one of comparatively slow progress, we ascend the scale of rapidity of fatal influence exercised by them through their powers of repeating themselves at a distance from the prime seat of disease, and so infecting the system at large. Epithelioma, which we frequently meet with at some part where mucus membrane and skin join—such as on the lips—is the least formidable of the true cancerous growths, and is the most amenable to treatment by removal with the knife or other agents. It is also a malignant growth which can be more clearly associated with distinct local irritation as a starting-point than any other of the series of cancers; for whereas, in the majority of the others, it is difficult to assign an apparent cause of the disease, in this one we can often plainly see what has set it up. In many a case the constantly-repeated irritation of the lip from smoking a short clay pipe with jagged edges has clearly induced an inflammatory swelling there, and after awhile this swelling has declared itself as an epithelioma. In the same manner cordwainers who draw strings through their lips have often suffered from the disease; and sweeps who have allowed soot to collect elsewhere, from want of attention to personal cleanliness, have been subject, as a class, to the same disastrous ailment. And, for my own part, I would say that, reasoning by analogy, we shall come to regard cancers generally as resulting from some stimulation of growth of cell-life from irritation, with, in all probability, some special imprint through infection from outside the system. I would have you know that the disease is not confined to human beings and to animals; it is well known to affect trees and plants, which it kills by interfering with their growth and by diffusing a poison through them, as it does in us. I find, on turning to the "Medical Annual" of this year, very interesting references by Mr. Keith W. Monsarrat to the observation of this subject by Dr. Noel Nason, who has brought forward most suggestive facts about the existence of districts where cancer seems to be unduly prevalent. It is there stated that "the idea of a connection between arboreal and human cancer . . . originated in the observations of the natural features of cancer districts, and the study of malignant vegetable tumours found in woods and orchards. Not only do 'cancer houses' abound in such districts, but individuals whose avocations bring them into these surroundings show a cancer proclivity above the average. . . . These vegetable tumours are apparently contagious, as several usually exist on neighbouring trees." What makes me more and more believe that we shall in the end find infection to be the prime cause of this disease is that the more close investigations are made into the nature of illnesses which follow definite lines in their development, the more often we are rewarded by discovering that they originate in this way. Take tuberculous diseases as an example of this fact. Until fairly recently they were regarded as being inherited and as being in no way contagious or infectious. The lectures of no less an acute observer than Sir Thomas Watson, published in 1848, speak of tubercles as being "composed of unorganised matter deposited from the blood," whereas they are now known to be the products of infection by a micro-organism which floats about in the atmosphere, having got there from previously infected persons. Sir Thomas answers the question, "Is phthisis contagious?" by saying "No; I verily believe it is not. A diathesis is not communicable from person to person. Neither can the disease be easily (if at all) generated in a sound constitution. Nor is it ever imparted, in my opinion, even by one scrofulous individual to another."

Just as these views, in the light of later knowledge, are now ascertained to be incorrect, I firmly believe that patient investigation will reveal to us that cancers are due to infections. But at present we know nothing of the sort, and are compelled to say that the origin of these malignant diseases is veiled in obscurity. Turning now to the clinical side of the question, I will tell you of the way in which malignant growths manifest themselves. They appear as swellings in the parts they have invaded, to which attention is soon drawn by the pain which follows their appearance. Hardly any region of the body is free from liability to cancer, but some organs are never invaded by it in a primary form—that is, it never originates in them, but only obtains a footing there by transference from elsewhere. A peculiar feature of the tumours which form in the first instance is the one I have already mentioned—that of their attachment to the parts they lie in. If we examine a true cancer of the breast, for instance, we shall find that the tumour we are handling does not move freely, but that it is adherent to the adjoining structures. And we shall probably find, on examining the armpit, that some glands there are enlarged, and that they are painless. If left to itself the tumour will continue to grow more or less rapidly and will affect the skin, causing the latter to be adherent to it in the first place, and finally to ulcerate. In cases of scirrhus, or hard cancer, the skin will pucker in and the nipple will be drawn in, even to disappearance; in the softer forms of the disease it will become thick and swollen before it breaks into an ulcer. Fortunately we do not often see such bad cases as these nowadays—not, at any rate, unless at a recurring stage—for it is the invariable practice of good surgeons to urge an early removal of the tumour as soon as is possible. We can seldom, however, see people before the glands in the neighbourhood have become infected by a secondary deposit of the cancer cell; hence we do not get a good chance of eradicating the disease by the knife. It is being rapidly recognised that an operation which only aims at removing the primary tumour is incomplete, and that it is necessary to remove all enlarged glands and the fatty tissues which lie in their vicinity, if we are to do any good. Even then it is greatly to be feared that we have only dealt with secondarily affected parts which have been accessible, for these glands have absorbent vessels and other glands within the body out of the reach of the surgeon's knife. Still, an increasing measure of success is attending the very bold and thorough surgery of the present time, in which a clean sweep is made of all absorbent tissues which can be reached by us, and there is no doubt whatever but that life is very considerably prolonged by these radical operations. What too often happens, however, is that the cancer cell has been absorbed, and that having been carried to some internal organ, such as the lung or the liver, it has lodged there and has set up a secondary infection in its new habitation; in such a case the disease makes rapid progress, the infected organ enlarges rapidly, and symptoms, due to local disturbance and to general infection quickly declare themselves, and the sufferer quickly succumbs. The especial thing to remember about the various malignant diseases is their tendency to recurrence either at their first seat or elsewhere. What, then, will be your duties in attending any sufferers by this dire disease? They will be those of trying in every way that lies in your power to soothe mind and body. The former you endeavour to effect by a cheerful demeanour, and by your kind and constant sympathetic attendances; the latter by carrying out to the full the directions as to dressings given you by the attending surgeons. You may be called upon to use deodorant and antiseptic preparations very freely to rob the cancerous ulcers of their offensiveness; and in cases marked by pain you may be ordered to give large doses of pain-quelling drugs—for pain, in many cases, is of a very severe character, and can only be kept under by the freest administration of anodynes.

### The American Hospital Ship.

By our Commissioner.

When I joined the special train which left Fenchurch Street at 1.15 on Saturday morning, taking a large company of distinguished visitors belonging to both the English and American nations to the West India Docks, with the object of inspecting the hospital ship "Maine," I noticed that, in spite of the bustle of, in many cases, a very hurried departure, the conversation was chastened in character. The gravity of the news of the repulse of General Buller at Colenso cast a shadow over the company, subdued the spirits of the most talkative of the ladies, and restrained the propensities of the most inveterate joke-makers among the men. It was, perhaps, a relief to many that just before the train moved two friends who had read of the escape from Boer custody of the gallant son of Lady Randolph Churchill, were heard on the platform to propound, in the shape of a puzzle, the question, "Where is Winston?" At least the passengers in my compartment, who were nearly all Americans, took up the topic with avidity, and fervent were the wishes expressed, especially by the younger ladies, that the escape effected might not be followed by another capture.

### On Board the "Maine."

There was no incident on the brief journey down, except the sudden and welcome appearance of the sun, which shone with something of the warmth of early spring; but many were the exclamations of disgust at the dirty, dark, depressing shed which is considered sufficient for the public who have to alight at the West India Dock Station. Moreover, if the way to the vessel was not long, it was excessively greasy, and if the prevailing fear of fog had been realised, some of the visitors might not have relished their experience. But in pleasing contrast were the tasteful decoration of the docks, the genuine enthusiasm of the spectators, and the admirable arrangements of the police. Every care had also been taken to provide as far as possible for the comfort and convenience of the invited guests, who quickly but quietly crowded on to the promenade deck and began to try and look about. This was not easy. The Duke and Duchess of Connaught, with the Princess Louise, Marchioness of Lorne, had arrived by a previous train, and having made their inspection of the ship, were at lunch with Lady Randolph Churchill, Mrs. Ronalds, Mrs. Blow, and other members of the committee; and there was a period of waiting for the ceremony of the day—the presentation, on behalf of the Queen, of the Union Jack to the vessel.

### The Wards of the "Maine."

Of this pause I took advantage to slip down into the wards, and, thanks to the courtesy of one of the officials, gleaned practically the information I sought. Much remained to be done, but here, as in all other parts of the vessel, the work of converting a cattle boat into a hospital ship has been most successfully carried out. So far as light and air are concerned, the wards leave nothing to be desired. By means of the heating arrangements and the use of a plentiful supply of electric fans, the temperature need never be varied, whatever the climatic condition may be. Personally, I should prefer to be nursed in the "Britannia," the "Columbia," the "Whiteland Reid," or the "Bernard Baker," but the committee isolation ward is inferior only in size to any of the other four. The beds, of which there are 218, are not placed too closely together, single cots being reserved for the more serious cases, and among many evidences of the anxiety to take good care of the patients is an ingenious contrivance of sliding curtains to protect them from draughts. The floor between the beds is carpeted with rubber canvas, which is noiseless. Neither effort nor expense has, apparently, been spared to make the operating chamber as perfect as circumstances will permit. The Röntgen Ray apparatus is a valuable feature. Of course, electricity is the motive force of the light throughout the vessel, and bells from each ward are in communication with the cabins of the doctors and of the superintendent nurse. The quarters for the nurses—which include a small saloon—and for the doctors are sufficiently capacious, and are excellently planned.

### The Scene on the Quarter Deck.

Returning to the promenade deck, I was merely able to get a glimpse of the houses which have been put up for the occupation of the military commander of the "Maine" and Lady Randolph Churchill respectively, and are furnished tastefully though not luxuriantly. In fact, there has been an avoidance of luxury throughout. The sum of £35,000, which it is understood covers the cost of the undertaking, has not been frittered away in superfluities. There is all that is essential on the "Maine," from the steam kitchens to the topmasts; but there has been no waste. Lord Charles Beresford, who was warmly congratulated upon his appointment to the Mediterranean command, was foremost in expressing his approval of the equipment of the ship. Accompanied by Lady Charles, the popular admiral was one of the most conspicuous of the group immediately surrounding the Duke of Connaught on the quarter-deck, where his Royal Highness delivered the speech preliminary to the presentation of the Queen's gift. The scene was one which those who witnessed it are not likely to forget—it is already historic. In addition to eminent visitors from town, the Cabinet being represented by the Lord Chancellor, were the entire staff of the ship, Miss Hibbard and her colleagues, with their blue dresses relieved by the red cross badge and white caps, forming a capital foil to the surgeons and male nurses in their uniforms of black and gold. The Church Lads' Brigade acted as a guard of honour.

### The Presentation of the Flag.

If the costume of the Duke of Connaught, who was attired in the ordinary dress of an English gentleman, was not picturesque, his manner was as impressive as his words were well chosen. Commencing with what was, in effect, a message from the Queen, anxious to convey her appreciation "of that charity which a large number of American ladies and gentlemen have shown towards the welfare of her kin, speaking their own language, who are now fighting in South Africa," he went on to signify his own pleasure at being asked to perform such "a unique ceremony." "Never before," he said, in clear tones audible to everyone, "had a ship sailed under the combined flags of the Union Jack and the Stars and Stripes." Perhaps no portion of the brief address more appealed to his remarkable audience than his assurance, "as an officer of the English army," of the deep gratitude which he felt convinced would be entertained by the officers and men who were to reap the advantage of "the splendid present" that had been made in aid of our wounded.

### Lady Randolph's Response.

When the cheers which followed the close of the speech had subsided, Lady Randolph Churchill, whom the Duke directly mentioned, offered, in an unfaltering voice, the thanks of the committee to Her Majesty for her gracious gift, and felicitously expressed her hope that the "Maine" will be more than useful "on her errand of mercy."

The religious part of the ceremony did not take many minutes, but it was by no means perfunctory. The Bishop of Islington, clad in his episcopal robes, dedicated the flag, and the ship over which it floated, to the service of God, and having also invoked a sacred blessing on the American and the Red Cross flags, the three were raised and unfurled—the

Union Jack, with the united arms of St. George, St. Andrew, and St. Patrick, and in the centre, on a white medallion, the red Geneva cross, on the main mast; the Stars and Stripes at the mizzen; and the Red Cross on the fore mast. Loud and prolonged cheers were given as each flag was put into position, the Scots Guards playing "Rule Britannia," while the Union Jack was run up and "The Star-spangled Banner" while the Stars and Stripes got aloft. Then, of course, the band struck up "God Save the Queen," and the memorable ceremony ended.

### The Return to Town.

Happily, in spite of a considerable crush, there was no accident. But an enterprising lady, in her eagerness to obtain a point of vantage on the roof of the deck cabin, missed her footing, and, but for prompt assistance, would have been precipitated into the water. The Royal party did not leave the vessel immediately after the presentation of the flag had been made, but remained for some little time conversing with the officials and others. Ladies observed that the Duchess of Connaught was seasonably clad in a warm cloak of black silk reaching to her feet, which was brightened by a glittering yoke, and made to look cosy by the addition of some beautiful fur. Her toque, a judicious mixture of soft sable and white flowers, was particularly becoming. The special was timed to return at 3.50, but, finding that the Royalties were going back to Fenchurch Street an hour earlier, I managed, with a few others, to secure a seat in the train reserved for them, the result being that on our arrival at Fenchurch Street we were, much to our amusement, supposed to belong to the Duke's suite. But, as one of the ladies said, "What is the use of hovering about Royalty unless you share their advantages?"

### The Farewell Dinner.

On Sunday the farewell dinner to the surgeons, nurses, and officers, for the benefit of the Hospital Ship Fund, took place at the Carlton Hotel, that evening being fixed because the directors, who gave the dinner, could not arrange for so many tables any other night. They also provided the elaborate decorations, and the whole of the rooms on the ground floor were placed at the disposal of the Ladies' Committee. The guests, about three hundred, included the Duke and Duchess of Connaught, the American Ambassador and Mrs. Choate, the Marquis of Lorne, and Lady Randolph Churchill, who sat at the central table. There were no toasts, and therefore no speeches; but as soon as the banquet was over the company adjourned to the reception room, where a concert by American artists was given. Owing to some of the tables being sold by auction, the amount realised by the dinner was no less than £2,000.

### The Princess Christian Hospital Train.

On Wednesday Princess Christian, accompanied by a small party including Lord Wantage and Sir John Furley, travelled to Birmingham in order to inspect the Princess Christian Hospital Train, which is shortly to be shipped to the seat of war. This, the first hospital train ever built in this country, consists of seven bogie corridor carriages. In the first are compartments for stores, two nurses, and two injured officers. The second contains accommodation for two medical officers, their surgery, and a dining-room, while in four others there is suitable provision for 18 patients. The seventh is occupied by the kitchen and pantry, and four hospital orderlies.

### The Gifts of the Royal Family.

Every member of the Royal Family has personally helped to make the train in all ways a success. The Queen herself has contributed a supply of excellent blankets, and there is a good collection of cushions and of little luxuries and comforts of all kinds sent either by Princess Christian or by other members of the Royal Family. Princess Christian, moreover, supplemented the £6,100 raised by the borough of Windsor towards the cost of the train by a donation of £650, the balance of a Red Cross Fund which was invested in Her Royal Highness's name after the Soudan campaign of 1885. The Princess has also taken a most active part in regard not only to the general idea, but in respect to the smallest details.

### The Wards.

As already explained, the fundamental object of the train is not merely to provide a means of conveying the wounded from the front to the coast, but to have a perfect self-contained hospital on wheels, so that if it should be shunted on to a siding, or otherwise get blocked, for even a week at a time, the wounded will be just as comfortable, and just as well nursed, as if they were in hospital. The beds are in three tiers—one on or quite close to the floor, one in the centre, and one near the roof. The difficulty of placing helpless invalids sideways on beds completely enclosed except on one side, with a narrow gangway to operate in, has been adroitly overcome by an arrangement of pulleys fixed in the roof, by means of which one attendant can raise, or lower, each bed to the desired level, while another guides and pushes it into position.

### The Fittings and Stores.

The cooking appliances are a marvel of completeness. There are several cisterns for cold water storage, supplemented by two large filters, and there is a refrigerator which will ensure the freshness of the meat. Of linen, clothing, spring wire mattresses, surgical and medical stores, cutlery, glass, crockery, and provisions there will be an ample supply. A very cheerful effect is produced by the enamelled white ironwork of the train and its fittings, and by the bright draperies. Externally, the centre panel on each side of every carriage has a conspicuous red cross painted on white ground, with the words "Princess Christian Hospital Train" in royal blue and gold. In sockets at the head of the train are the Union Jack and the Red Cross flag, in accordance with Article VII. of the Convention of Geneva.

### The Staff.

Early next month Sir John Furley, the creator of this most complete hospital train, will go to the Cape as Special Commissioner of the Central British Red Cross Committee in order to see that his "child" fulfils all the objects which it is intended to serve. In addition to two surgeons and the officers of the St. John Ambulance Brigade, two members of the Army Nursing Reserve—Miss Creighton and Miss Jones—will accompany the train, which will be packed in sections in large cases and reconstructed on its arrival at the Cape, to be used wherever it is required.

### Death in Our Ranks.

Another Case of Typhoid.—With much regret we have to record the death of Miss Bertha Foster from typhoid fever at the Derbyshire Royal Infirmary, in the second year of her training. Miss Foster was, says one who knew her well, a most kind and gentle nurse, a general favourite amongst her fellow probationers, and her early death is greatly deplored.

### Christmas in a Fever Hospital.

By One of the Nurses.

The ward was much like the wards of other hospitals. It contained two long rows of beds, whose occupants appeared to be in a more or less acute stage; some tossing restlessly, and others sitting up in bed to watch the progress of the decorations. Two nurses were fixing some sprays of holly over the door, and a third was arranging other sprays for fixing, with the help of a convalescent patient, a weakly-looking child of 10. The latter were kneeling at their work, and as they worked they chatted. "Molly," the nurse was asking, "what keeps you awake in the night? a healthy convalescent"—with a doubtful glance at the rickety limbs—"should sleep soundly; but night nurse says she often finds you crying."

"Oh! that's ony of foggy nights, Nurse Marion; you see I'm kep that busy in the dye, tykin' messages from one patient to another, that I forgets to be sorry enough about Meg's cough—Meg and me is twins you know—an' the doctor sez she's got consumption; an the fogs catches 'er breath that 'ard, you'd think she'd die coughin'. Well, in course, when I'm at 'ome in bed wiv 'er we 'ugs each uvver tight to get warm; an' when we're warm at the front, there's little wriggles of cold all down our backs, so we tykes it in turn to roll over to get our backs warm; an' it makes me cry to fink that Meg is gettin' all the wriggles to 'erself now. Sy! nurse, is there any charnst of us getting' anyfink give to-morrer? like skirts an' perticits, or a night-gownd for a chynge?"

"No, Molly," nurse said sadly, "and I'm afraid that much more is needed at your home than one warm night-gown."

"But," argued the child, "we should both tyke it in turns to wear it; we're pals, is Meg an' me! If lydies wuz to send 'em would Mytron let us tyke 'em 'ome wiv us? We ain't ter tyke our toys 'ome."

"Certainly she would," nurse replied, "because she would store them in a safe place until you left us; but, of course, the toys which have been amongst the infection must remain to amuse the next batch of sufferers. But charitably-disposed people cannot visit these hospitals, so they are apt to forget that the chief factor in the spread of infection is want, and to feel that their duty towards them is done when their rates are paid."

There was a pause, broken by Molly, who said, "Mrs. Johnson's put on the danger list, ain't she? 'Er 'usband cyme this afternoon. My eye! but 'e'd tyken more 'en a drop to keep 'isself from ketchin' the fever. That's 'er a-callin' 'Nurse' now."

Nurse Marion rose, and crossing to the bedside of an emaciated, weary-looking woman of about thirty-two, said, "What can I do for you, Mrs. Johnson?"

"Can't you come an' sit with me a little while, nurse; it's 'orrid lyin' by myself, thinkin' an' thinkin'."

Drawing up a chair, and taking one of the thin hands in her own, nurse asked, "What are you thinking and thinking about to worry you, dear?"

"Oh, everythink, nurse—my baby, wot my landlady minds while my eldest girl's at 'er work—though she's not a bad sort, isn't Mrs. Brown, for she ony gets drunk of 'olidays, an' Mary is 'ome to mind it then. But, 'specially, I'm bothered 'bout myself since my 'usband told me the doctor 'ad writ to say I'm worse, an like ter die. Not 'at I'm afeard of death, fer' myself—fer it can't be worse an' life is, when food is scarce, an' the knawing 'unger yer feels in yer own vitals ain't nothink agenst the gripin' 'unger yer feels for the five empty stomachs at 'ome.

"Well, I wuz warned as, if I married Flash Charlie, I should lie on a 'ard bed of my own makin'; but it's bin 'arder an' flint 'as the bed, an' my own flesh an' blood 'as 'ad to share it, too—which it's a 'ard price to pay for bein' too young to understand. For a time my man did 'is best—which 'is best was bad enough—an' until my third wuz born, we never starved, an' alwuz got meat 'on Sundays. But 'e's been drinkin' an' out of work ever since; an' the children came that quick as I 'adn't strength to give 'em all; an' between times charin' was not reglar, and not alwaz ready-money. So Jim, my eldest, was so tiny at thirteen 'at 'e couldn't get took on nowhere—an sellin' papers isn't wot it used to be; it's overstocked is the trade, an' the big boys used to steal 'is papers, an' pull 'is 'air when 'e cried. But 'e struggled on; an' 'e's a 'ero to me is our Jim, though people will cry 'goal-bird' after 'im all 'is days, for 'e's doin' two years in a reformitry.

"My Mary did well fer 'erself; fer, though she's on'y twelve, she looks sixteen, an' she earns four shillin's a week washin' jars at a jam factory. Still, 'er wage wasn't due till the Friday, as we were a-took bad on the Thursday—Tommy an' Minnie an' me. Tom an' Min wuz moanin' an' cryin' like anythink; an' Mary 'ad come 'ome with toothache an' was bangin' 'er 'ead agenst the floor, which brought Mrs. Brown up to arst wot the row wuz about; an' she advised me to send for a pennorth of sweet nitre to sweat it out of us, an' a pennorth of laudnaum for Mary's tooth.

"Well, we'd ony tuppence left, an' nothink as we could pawn; and I knew as we must 'ave a tuppenny loaf to keep the workers agoin', so I said as 'ow 'praps we should all feel better in the mornin'. But, while I wuz sayin' it, I came over all giddy like, an' dropped all uv a 'eap, an' I must uv fell asleep, fer, when I opened my eyes, there wuz a pliceman in the room, an' my poor Jim 'ad 'ancuffs on 'is wrists, an' on the table wuz two little bottles, one marked 'sweet nitre' an' the other 'laudnaum'; an' agenst 'em wuz a tuppenny loaf.

"'Wanted fer pickin a till,' sez the pliceman, an' my Jim only sez, 'If I'd a-took silver, instead of the coppers, they wouldn't a-heard the chinkin' in the back room; but I didn't want to be a bigger thief 'an I could 'elp.'

An' the next time I opened my eyes I wuz in this ere bed; an' nurse she tell me 'at Tommy, 'an Minnie, wuz took to the South Northern Orspitle coz this un wuz full up; an' I 'ear the're a-goin' to be discharged next week.

"But I means to keep on 'opin', nurse; an' please God, I gets over this; an', please God, I keeps a roof over our 'eads fer my boy when 'e leaves 'is prison gates, then I'll praise 'im orl the days of my life."

### In a Christmas Library.

LADY BARBARITY. (Ward, Lock, and Co., Limited.)

"THIS is the name the beaux had dubbed me, because, said they, you are so cruel," is the explanation which Mr. Snaith's heroine gives of her title of "Lady Barbarity." The fair egotist tells the reader that she wearied of her triumphs in town, and in the winter of 1746 she returned to her home in the North, and to "my papa the Earl." The latter has recently learnt that he is not likely to live long, and he receives the doctor's verdict with the excellent courage which is his only redeeming trait. In an interview with his daughter on the day following her arrival, the father refers to his own condition, and tells her that he shall not be content unless he leaves her at her ease, and advocates a marriage—"make yourself a duchess, with a hundred thousand pounds." A rebel, in the custody of Captain Grantly and eight men, arrives on the scene, and thus the element of romance is promptly introduced. The captain is one of the lady's 'hundred triumphs," and her attempts to utilise his admiration for her own ends are worthy of a better cause. The prisoner makes his escape by the aid of this very indiscreet young lady, and this brings about serious complications of a more or less improbable character. The rebel's chief attractions appear to consist of a pair of fine eyes and an unlimited stock of impudence. In the end he wins Lady Barbarity's affections, shoots the unfortunate captain, cheats the gallows at the very scaffold, and the marriage which eventually takes place brings an unsatisfactory story to an unlikely conclusion.

HER OWN WAY. (The Religious Tract Society.)

A GENTLE mother, two strong-minded daughters, and their pretty, high-spirited half-sister, Juliet Tracy, form the family round which Miss Eglantine Thorne has woven this story. Hannah and Salome have small sympathy with their spoilt junior, and the mother is powerless to influence the child, on whom she lavishes unlimited love. Juliet, contrives to get into a very serious scrape, but escapes with a useful but painful lesson as to the peril of taking her own way.

A DAUGHTER OF FRANCE. (Thomas Nelson and Sons.)

THIS is another historical and adventurous story treating of the stirring times of the persecutions of the Huguenots and of survivors of "La Rochelle." The English and French who left their own lands to gain in a new country the religious freedom denied them at home are well described. It is strange to read of Boston as a village, and Miss Eliza Pollard has depicted many harrowing scenes and given us an acquaintance with contemporaneous politics. Nova Scotia, Arcady, and Canada form the extensive backgrounds for the Indians, Jesuits, and Huguenots, whose fortunes and misfortunes are presented to the reader.

DRIFTWOOD. (The Religious Tract Society.)

IN this novel Miss Mary E. Palgrave gives us the history of a brother and sister who are plunged into poverty on the death of a not particularly judicious father. Sally, the old nurse, belongs to the type of family retainer, and her devotion to her "bairns" is great, although in the case of Marjory it goes to the length of aiding her in escapades of the most indiscreet description. Oliver is a worthy, uninteresting young man, whose unsympathetic disposition renders him the worst possible companion for his emotional sister. His harshness is almost brutal at the time of his father's death, when he treats the depressed young girl in a very heartless manner. The development of the two characters and their eventual conversion to better things is sufficiently interesting. The wife whom Oliver chooses is perhaps the best drawn and most lifelike study in the story.

THE RED MEN OF THE DUSK. (C. Arthur Pearson, Limited.)

THIS is an exciting romance, in which most of the scenes are laid in Wales, the hero being a Worcestershire man. The events which he records are represented as his personal adventures in the spring of 1660, and they are of a nature to delight the schoolboy who loves to read of much slaughter and bloodshed. There is plenty of fighting, and the hero has a delightful mare called Whitesock, who distinguishes herself and succours her master in various ways. Mr. John Finnemore introduces us to a fascinating heroine and one or two other spirited women, and in spite of the gunpowder atmosphere the story ends happily.

AN INCORRIGIBLE GIRL. (The Religious Tract Society.)

THERE is power and freshness in this story of M. H. Cornwall Leigh's, and the very incorrigible Lydia is interesting in her many variable moods. The scenes in the country, through which the girl wanders in search of wild flowers, are charming, and it is obvious that the authoress is an observant lover of nature. But a jarring note is introduced with the institution known as the Rescue Home, or County Council Experiment. It is difficult to know the object of this wholesale condemnation of a Home. The bad management, favouritism, and cruelty make unpleasant reading, and if the facts are true as regards any modern English institution the sooner they are proved the better for the nation's reputation. The petty persecutions and the decree of the matron that Lydia should be kept indoors for months form part of a system more in accordance with the beginning than the end of the nineteenth century. But there is a happy future reserved for Lydia, who becomes the confidential nurse-companion of an adorable young lady with whom we leave her comfortably established in a becoming imitation of a trained nurse's uniform. Her excellent brother, who has endeavoured to be at all times as respectable as his incorrigible sister permitted, is satisfactorily disposed of in a training ship. The authoress certainly shows that it is easier to make a decent citizen out of a boy than a girl, but then Tom was an exceptionally promising example.

HAVELOK THE DANE. (Thomas Nelson and Sons.)

THE legend of Old Grimsby and Lincoln which Mr. Whistler has given us in the form of a capital story is one of the best which we have read for some time. It is full of a breezy kind of vigour which is immensely refreshing. The preface gives particulars as to the sources by which the legend has been handed down. The heroes, both Danes and English, are of a strong individuality, which commends them to the understanding as well as the affections of the reader. We lay down the fascinating volume with a regretful feeling at having to say farewell to Havelok, Sigurd, and Queen Goldberga. The book has been illustrated, with great success, by W. H. Margetson.

A CAPTAIN OF IRREGULARS. (Thomas Nelson and Sons.)

THIS is a really handsome gift book and likely to be as popular as Mr. Herbert Hayens' other stories, and the illustrations are delightful, Sydney Paget being the artist whose clever pencil has portrayed the warriors of the beginning of the century. Life and many adventures amongst Spaniards of both sexes keep the reader's interest keenly sustained from the first page to the last. The "warhorse" Piray supplies a specially exciting moment, but is eventually mastered by the English lieutenant who "can ride a little bit," and to whom he proves a saviour in the future. The dialogue is very bright, and the whole story brings us into very close contact with the realities of war.

### Echoes from the Outside World.

AN OPEN LETTER TO A HOSPITAL NURSE.

CHRISTMAS is close upon us, so close, indeed, that another three or four days will see the dawn of that morning which should bring with it "peace on earth and goodwill towards men." But, alas! it finds us overwhelmed with the sorrows of a great war. Yet "overwhelmed" is hardly the word to use, for though during one brief week three serious reverses have befallen our arms, and our casualties—officers and men killed, wounded, and missing—have almost been doubled, there is no suggestion of panic amongst us. The ill tidings have only made most of us, even the women, who have to give their dearest, fully determined that more men must be sent, more lives must be hazarded, to help our native land. To those who are fairly young the saying that "an Englishman never knows when he is beaten" has been only a tradition, but now its truth is being once more emphasised in grim earnest.

SOME of the foreign papers which have been friendly to the Boers all along treat us gently in our trouble, one admitting that "the tranquility with which England receives news of the misfortune forces from us respect for their nation." Thus, a Viennese organ says, "England's mightiest power is her command of grief. In every other State, in face of such events, the people, with cries of rage, would have risen up against the Government. England's dignity and composure are not lost for a moment. This is real greatness in a people, and signifies England's greatest strength." But that quietness and calmness may go hand in hand with action and enthusiasm is being abundantly proved by the rush of the volunteers, who are asked, not commanded, to give their services. The intimation that the Government would welcome offers from the volunteer forces was only known on Monday morning, and yet everywhere, all day long, men were giving in their names as quickly as they could be written down, and the numbers still continue to increase.

THE announcement that Lord Roberts and Lord Kitchener are going to South Africa has created a great impression. Not as some of the horrid papers, suffering as much from ignorance as from want of good feeling, stated to "supersede" Sir Redvers Buller, but because a larger army needs a more important head. When a small force was holding Natal, a major-general (General Symons) held command, but as soon as the number of troops exceeded that with which a major-general is usually entrusted, a lieutenant-general (Sir George White) took supreme command. He in turn was followed by a general (Sir Redvers Buller), and now as there is to be a large increase in the forces, an officer of yet higher grade is appointed, a field-marshal. Had there been any question of supersession a general of his own rank would have taken General Buller's place, but the despatch from the War Office exactly explains the situation, that "as the campaign in Natal is, in the opinion of Her Majesty's Government, likely to require the presence and undivided attention of Sir Redvers Buller, it has been decided to send Field-Marshal Lord Roberts to the Cape as Commander-in-Chief in South Africa, with Lord Kitchener of Khartoum as his Chief-of-Staff." The Queen is evidently anxious that her people should feel she is one with them in their grief and anxiety, or she would not have announced her intention of remaining at Windsor for Christmas. It is difficult to remember the time when she did not go to Osborne for the festive season, and disappointment at her decision is sure to be great in the Isle of Wight; but the nearer she is to London the easier and the more quickly she can be consulted on matters of importance, and one cannot but be grateful to her for her thoughtfulness and for the sacrifice of her own desires. This has been again exemplified by her proposal to entertain a large number of the wives and children of the soldiers serving in South Africa at Windsor Castle on Boxing Day. There are to be grand doings in St. George's Hall, where, after tea, presents to the women and little ones will be distributed off a Christmas tree 25 ft. high. All expenses will be defrayed from the Queen's private purse.

IT is not only in Royal circles that misfortune is giving rise to manifestation of nobility and unselfishness. Our heart aches for poor Lady Roberts, learning almost at the same moment that her only son had died of his wounds received at Colenso, and that her husband is ordered abroad. The Earl of Dudley has offered his services to the Government, and has the promise of many members of the Dudley Squadron of Worcestershire Hussars to accompany him, whilst Lord Chesham is getting together a force of 3,000 Yeomanry Cavalry for service in South Africa, whom he will command in person. Then the Duchess of Portland, several days before she received the news of the death of her only brother, Lieutenant Dallas Yorke, from typhoid, had announced that there would be no entertainments this winter at Welbeck Abbey, because she felt rejoicing would be out of place whilst so many were sorrowing, and that the money would be needed by the sick and suffering. Sir William Ingram has withdrawn all his horses from training on account of the war. He usually spends about £25 a week on his stables. This sum will be sent to the Widows and Orphans' Fund as long as the war continues. Lord Hopetoun—the Lord Chamberlain—has been in the habit of giving a yearly ball to the servants and employés on his estate, but this year it has been decided that it shall not take place, because so many who would have attended have lost relatives in the Highland Brigades. Therefore, as he is unwilling to be a pecuniary gainer, £75, the estimated cost of the entertainment, has gone to the Widows and Orphans' Fund. The Lord Mayor, I see, has sent £100 by cable to the City of London Regiment, the Royal Fusiliers, as a Christmas gift, and even amongst private individuals I hear of many who are not having their usual social gatherings, and who are sending no Christmas cards, in order to be able to contribute their mite to the War Fund. But perhaps no case of individual self-sacrifice is more interesting than that mentioned by Mr. Goschen of the retired naval officer who, with limited means, placed his retired pay of £400 a-year at the service of the Government for such object as they thought might best conduce to relieve the necessities of the day.

THE case of Louise Masset has attracted great attention, partly because she availed herself of the privilege of giving evidence on her own behalf, and partly because of the deliberate manner in which the crime of the wretched woman appears to have been planned. It is impossible to doubt her guilt, and for a mother who, without, as far as can be gathered, the slightest touch of insanity, kills her own child, a bright little boy of three, there can be no possible plea of mitigating circumstances. Louise Masset's only motive—and that was denied—was the desire to get rid of an incubus which might interfere with a disreputable *liason*. At the same time, the execution of a woman for the murder of an illegitimate child has been so rare during the fast-waning century, in spite of many convictions, that it is thought the murderess in this instance will not suffer the extreme penalty of the law. Most of us, I hope, will rejoice if the sentence is changed to one of penal servitude, for the hanging of women seems worse than that of men; but a respite must, none the less, have the effect of strengthening the movement for the abolition of capital punishment.

### The Princess of Wales's War Fund.

It is with much pleasure that we publish the first encouraging result of the announcement that we have consented to receive subscriptions to the Princess of Wales's War Fund. All subscriptions should be addressed to the Manager, Office of The Hospital, 28 and 29, Southampton Street, Strand, London, W.C., and name and address should be given.

| | s. | d. |
|---|---|---|
| Mr. Louis H. M. Dick | 5 | 0 |
| Edith Machin ... ... | 5 | 0 |
| A. B. Potter ... ... | 5 | 0 |
| Nurse Wratten ... | 5 | 0 |
| Nurse Mary Ashford, P.F. ... ... ... | 5 | 0 |
| A. Burn Bailey ... | 5 | 0 |
| Mary Hill, P.F. ... | 5 | 0 |
| May Hillkirk ... ... | 5 | 0 |
| H. M. Cowdell, P.F.... | 5 | 0 |
| S. A. Savile, P.F. ... | 5 | 0 |
| D'Arcy Adams, P.F.... | 5 | 0 |
| Jane A. Gealey, P.F.... | 5 | 0 |
| Sister Nightingale ... | 5 | 0 |
| Ada Oxley, P.F. ... | 5 | 0 |
| Grace E. Freeman, P.F. | 5 | 0 |
| Emma Butler, P.F. ... | 5 | 0 |
| Nurse M. A. Bell, P.F. | 5 | 0 |
| M. J. Packwood, P.F. | 5 | 0 |
| Mary Skevington ... | 5 | 0 |
| K. Whitehead, P.F. ... | 5 | 0 |
| M. H. Lowdell ... | 5 | 0 |
| Ethel C. James ... | 5 | 0 |
| Nurse Shrive, P.F. ... | 5 | 0 |
| Alice M. Ware, P.F.... | 5 | 0 |
| K. V. MacIntyre, P.F. | 5 | 0 |
| H. F. Philbrick, P.F... | 5 | 0 |
| H. C. Richmond, P.F. | 5 | 0 |
| Helen Rose, P.F. ... | 5 | 0 |
| E. M. Roberts, P.F. ... | 5 | 0 |
| Sarah Allcott, P.F. ... | 5 | 0 |
| Hutchings ... ... | 5 | 0 |
| Joan M. E. D'Avanport, P.F. ... ... | 5 | 0 |
| E. M. Roberts, P.F. | 5 | 0 |
| Ellen Clarke, P.F. ... | 5 | 0 |
| M. Oak Kuig, P.F. ... | 5 | 0 |
| A. J. Atherton, P.F. | 5 | 0 |
| Eliza Penny, P.F. ... | 5 | 0 |
| Miss Deas, P.F. ... | 5 | 0 |
| E. S. Gayton, P.F. ... | 5 | 0 |
| H. B. Duncan, P.F. ... | 5 | 0 |
| T. E. Ward, Brussels | 3 | 6 |
| Alice Fulcher, P.F. ... | 3 | 0 |
| E. MacRae, P.F. ... | 3 | 0 |
| Clare Baker, K. Smith, and A. Smith, P.F. | 3 | 0 |
| E. M. Zapp, P.F. ... | 3 | 0 |
| Davenport, P.F. ... | 3 | 0 |
| Janet Harbour, P.F.... | 3 | 0 |
| M. M. R. Napper, P.F. | 3 | 0 |
| F. P. Cohen, P.F. ... | 2 | 6 |
| M. E. Barber ... ... | 2 | 6 |
| L. Wilkinson, P.F. ... | 2 | 6 |
| J. M. Ellis ... ... | 2 | 6 |
| Govier, P.F. ... ... | 2 | 6 |
| Mercy Bristow, P.F. | 2 | 6 |
| E. S. Chapman, P.F. | 2 | 6 |
| Catherine Dunn, P.F. | 2 | 6 |
| H. E. Hugall, P.F. ... | 2 | 6 |
| Louisa Isaac, P.F. ... | 2 | 6 |
| Ada Owens, P.F. ... | 2 | 6 |
| E. C. Reynolds, P.F. | 2 | 6 |
| Annie Slight ... ... | 2 | 6 |
| A. Helen Denny, P.F. | 2 | 6 |
| M. D. Bristow, P.F. | 2 | 6 |
| Alice Godwin, P.F. ... | 2 | 6 |
| Ann Rice, P.F. ... | 2 | 6 |
| Jessie Brooks, P.F. ... | 2 | 6 |
| C. M. Dutton, P.F. ... | 2 | 6 |
| Helen Scott, P.F. ... | 2 | 6 |

| | s. | d. |
|---|---|---|
| Kate Pitts ... ... | 2 | 6 |
| Alesandra P. Strachan | 2 | 6 |
| Emily Waller, P.F. ... | 2 | 6 |
| L. C. Knight, P.F. ... | 2 | 6 |
| Marion Finch, P.F. ... | 2 | 6 |
| Miss M. S. Healy, P.F. | 2 | 6 |
| Mabel L. Everard, P.F. | 2 | 0 |
| Sister Lucas ... ... | 2 | 0 |
| Adelaide M. Warren, P.F. ... ... ... | 2 | 0 |
| Kate Walton, P.F. ... | 2 | 0 |
| Jannette Bryne, P.F... | 2 | 0 |
| A. J. Rose, P.F. | 2 | 0 |
| Nurse Chaundy, P.F... | 2 | 0 |
| Nurse F. C. ... ... | 2 | 0 |
| A. Frances Fullager, P.F. ... ... ... | 2 | 0 |
| Mrs. E. Chapman ... | 2 | 0 |
| A. C. Hall, P.F. ... | 2 | 0 |
| Hay, P.F. ... ... | 2 | 0 |
| Belle Jones ... ... | 2 | 0 |
| Jane Welch, P.F. ... | 2 | 0 |
| Emily A. Accocks, P.F. | 2 | 0 |
| M. A. Plowman, P.F. | 2 | 0 |
| Calver ... ... ... | 2 | 0 |
| Isa M. Davidson, P F | 2 | 0 |
| L. J. Mason, P.F. ... | 2 | 0 |
| E. E. Jennings, P.F.... | 2 | 0 |
| S. Johnson, P.F. ... | 2 | 0 |
| Charlotte Carter, P.F. | 2 | 0 |
| T. M. A. Parsons, P.F. | 2 | 0 |
| Matilda Redish, P.F... | 2 | 0 |
| Wilcox, P.F. ... ... | 2 | 0 |
| M. E. Smith ... ... | 1 | 6 |
| Mary Griffin Ennis, P.F. ... ... ... | 1 | 6 |
| Annette Dowling, P.F. | 1 | 6 |
| Ellen F. White, P.F. | 1 | 6 |
| Mary C. Palmer, P.F. | 1 | 6 |
| Mary G. Scott, P.F. | 1 | 6 |
| Lilian King, P.F. ... | 1 | 0 |
| M. A. Underwood, P.F. | 1 | 0 |
| S. A. Flanagan, P.F. | 1 | 0 |
| E. Covell, P.F. ... | 1 | 0 |
| Ransome, P.F. ... | 1 | 0 |
| Win. Flintham, P.F. | 1 | 0 |
| M. J. Phelp, P.F. ... | 1 | 0 |
| Rhoda Andrews, P.F. | 1 | 0 |
| Louisa M. D'Arcy, P.F. | 1 | 0 |
| Kate A. Thorne, P.F. | 1 | 0 |
| Mary Holton, P.F. ... | 1 | 0 |
| H. M. Freeman, P.F. | 1 | 0 |
| M. Haynes, P.F. ... | 1 | 0 |
| Adele L. Church, P.F. | 1 | 0 |
| Annie Turner, P.F. ... | 1 | 0 |
| Flrnce. C. Rhodes, P.F. | 1 | 0 |
| J. C. Bassett, P.F. ... | 1 | 0 |
| E. J. Edwards, P.F.... | 1 | 0 |
| T. Sherwood, P.F. ... | 1 | 0 |
| Henrietta M. Walker, P.F. ... ... ... | 5 | 0 |
| E. M. Liddle, P.F. ... | 5 | 0 |
| Mary Thomas, P.F. ... | 5 | 0 |
| M. H. L. W. (H. Lambert White), P.F. ... | 5 | 0 |
| Elizabeth Armstrong, P.F. ... ... ... | 5 | 0 |
| Mary Berrette, P.F.... | 5 | 0 |
| H. Robinson, P.F. ... | 5 | 0 |
| E. A. Dann, P.F. ... | 5 | 0 |
| E. Burton, P.F. ... | 5 | 0 |

| | s. | d. |
|---|---|---|
| Nurse Incheden Webber, P.F. ... ... | 5 | 0 |
| Lizzie Paul, P.F. ... | 5 | 0 |
| Policy 1,365, P.F. ... | 5 | 0 |
| L. G. Shipley, P.F. ... | 5 | 0 |
| K. Bevan, P.F. ... | 5 | 0 |
| L. A. Longstaff, P.F. | 5 | 0 |
| E. Streak, P.F. ... | 5 | 0 |
| M. A. M. Gilligan, P.F. | 5 | 0 |
| Nurse Sarah Cole, P.F. | 5 | 0 |
| Nurse J. E. Starling, P.F. ... ... ... | 5 | 0 |
| Miss Brailey, P.F. ... | 5 | 0 |
| M. J. Cheesman, P.F. | 5 | 0 |
| E. M. Williams, P.F. | 5 | 0 |
| Jane McCosh Smith, P.F. ... ... ... | 5 | 0 |
| Clara May, P.F. ... | 5 | 0 |
| A. V. Atkinson, P.F. | 5 | 0 |
| Miss Lloyd, P.F. ... | 5 | 0 |
| Nurse Manley, P.F. ... | 5 | 0 |
| Lucy Weatherly, P.F. | 5 | 0 |
| Mrs. S. J. Weston, P.F. ... ... ... | 5 | 0 |
| L. H. Shepherd, P.F. | 5 | 0 |
| M. C. Trinder, P.F. ... | 5 | 0 |
| A. M. Hobbs ... ... | 5 | 0 |
| Nurse Wood, P F. ... | 5 | 0 |
| Nurse Knights, P.F... | 5 | 0 |
| Ruth Cumberland, P.F. | 5 | 0 |
| Nurse Bate ... ... | 5 | 0 |
| Nurse H. Ellen Patterson, P.F. ... ... | 5 | 0 |
| Nurse F. T. Westmore, P.F. ... ... ... | 5 | 0 |
| L. Wyld ... ... | 5 | 0 |
| C. B. Edmonds, P.F.... | 5 | 0 |
| Nurse A. Spring, P.F. | 5 | 0 |
| Edith M. Epps ... | 5 | 0 |
| Nurse E. Blake, P.F... | 5 | 0 |
| C. McCalman, P.F. ... | 5 | 0 |
| Helen Gibbs ... ... | 5 | 0 |
| Miss Gilbart, P.F. ... | 5 | 0 |
| Nurse Isa Howie, P.F. | 5 | 0 |
| Emily Robinson, P.F. | 5 | 0 |
| Emma Norman, P.F... | 5 | 0 |
| Nurse Kenwright, P.F. | 5 | 0 |
| Nurse Edwyn, P.F. ... | 5 | 0 |
| Margaret Burrows, P.F. | 5 | 0 |
| G. T. Mentin, P.F. ... | 5 | 0 |
| A. Clark, matron, P.F. | 5 | 0 |
| G. S. Taylor, P.F. ... | 4 | 0 |
| C. Doran, P.F. ... | 5 | 0 |
| E. Knight, P.F. ... | 4 | 0 |
| A. Lee, P.F. ... ... | 3 | 6 |
| B. M. J. Graeme, P.F. | 3 | 6 |
| E. Ellis, P.F. ... ... | 3 | 0 |
| J. M. Blumfield, P.F. | 3 | 0 |
| Mary Brooks, P.F. ... | 3 | 0 |
| Nurse Constance, P.F. | 3 | 0 |
| M. J. Bewlay, P.F. ... | 3 | 0 |
| F. L. Wilde, P.F. ... | 3 | 0 |
| G. Fletcher, P.F. ... | 3 | 0 |
| Mary Clermiston, P.F. | 3 | 0 |
| Nurse Thompson, P.F. | 3 | 0 |
| Sarah Rouston, P.F.... | 3 | 0 |
| Jane Garrett, P.F. ... | 3 | 0 |
| Emily Ketley, P.F. ... | 3 | 0 |
| H. M. Walker, P.F.... | 2 | 6 |
| Ellen E. Ashton, P.F. | 2 | 6 |
| M. Tonks ... ... | 2 | 6 |
| M. L. Shaddock, P.F. | 2 | 6 |
| Sister Marjorie, P.F... | 2 | 6 |
| M. Smallhorn, P.F. ... | 2 | 6 |
| C. M. Robinson, P.F. | 2 | 6 |
| Alice Winning, P.F.... | 2 | 6 |
| Nurse Whitford, P.F. | 2 | 6 |
| Mrs. Barr, P.F. ... | 2 | 6 |
| M. C. Henderson, P.F. | 2 | 6 |
| Bessie Shipley, P.F.... | 2 | 6 |
| C. M. Ruttledge ... | 2 | 6 |
| Mary Cowley ... ... | 2 | 6 |
| E. Sayce, P.F.... ... | 2 | 6 |

| | s. | d. |
|---|---|---|
| Ellen Sharwood ... | 2 | 6 |
| N. F. Hill, P.F. ... | 2 | 6 |
| E. S. Daines, P.F. ... | 2 | 6 |
| C. M. Archibald, P.F. | 2 | 6 |
| A. K. Lawry, P.F. ... | 2 | 6 |
| R. Neale, P.F. ... | 2 | 6 |
| Hannah C. Houghton, P.F. ... ... ... | 2 | 6 |
| Eveline C. M. Wright, P.F. ... ... ... | 2 | 6 |
| Nurse Wheeler, P.F.... | 2 | 6 |
| Florence Newton, P.F. | 2 | 6 |
| Annie Longford, P.F. | 2 | 6 |
| Maria E. Goff, P.F. ... | 2 | 6 |
| L. A. Wing, P.F. ... | 2 | 6 |
| M. E. Kearsey, P.F.... | 2 | 6 |
| C. J. Forrest, P.F. ... | 2 | 6 |
| Kate Jeffreym, P.F. ... | 2 | 6 |
| C. E. Gould, P.F. ... | 2 | 6 |
| H. Arnold, P.F. ... | 2 | 6 |
| S. Arnold, P.F. ... | 2 | 6 |
| F. Ordish, P.F. ... | 2 | 6 |
| Louisa Dubkley, P.F. | 2 | 6 |
| S. Silverwood, P.F. ... | 2 | 6 |
| Edith H. Porter ... | 2 | 6 |
| Policy 5,019, P.F. ... | 2 | 6 |
| M. C. Reynolds, P.F. | 2 | 6 |
| Sarah Henwood, P.F. | 2 | 6 |
| Elizabeth England ... | 2 | 6 |
| Nurse O'Donnell, P.F. | 2 | 6 |
| E. Botham, P.F. ... | 2 | 6 |
| E. M. Geach, P.F. ... | 2 | 6 |
| Miss Batchelor, P.F.... | 2 | 6 |
| A. M. Brittain, P.F. | 2 | 6 |
| A. M. Norman, P.F. | 2 | 6 |
| Janet W. Jeffrey, P.F. | 2 | 6 |
| C. M. Kempsell, P.F. | 2 | 6 |
| S. V. Mallinson, P.F. | 2 | 6 |
| Policy 1,763, P.F. ... | 2 | 0 |
| A. M. Browne, P.F.... | 2 | 0 |
| M. Strange, P.F. ... | 2 | 0 |
| J. R. King, P.F. ... | 2 | 0 |
| Martha Jaques, P.F.... | 2 | 0 |
| Sister Julia Tudor, P.F. | 2 | 0 |
| K. Patman ... ... | 2 | 0 |
| Helen M. Blake, P.F. | 2 | 0 |
| Fannie E. Manning, P.F. ... ... ... | 2 | 0 |
| B. Greensell, P.F. ... | 2 | 0 |
| Olive M. Holford, P.F. | 2 | 0 |
| Miss Turpie, P.F. ... | 2 | 0 |
| A Bridle, P.F. ... | 2 | 0 |
| E. B. Johnstone, P.F... | 2 | 0 |
| E. K. Tallack, P F. ... | 2 | 0 |
| E. M. Ellis, P.F. ... | 2 | 0 |
| Nurse Hyland, P.F. ... | 2 | 0 |
| Miss S. Balsdon, P.F... | 2 | 0 |
| Phillis Legate, P F. ... | 2 | 0 |
| A. Megarry, P.F. ... | 2 | 0 |
| Edith Jondon, P.F. ... | 2 | 0 |
| Nurse Ellis, P.F. ... | 2 | 0 |
| Nora Steward, P.F. ... | 2 | 0 |
| M Hawley, P.F. ... | 2 | 0 |
| C. M. Cousins, P F.... | 2 | 0 |
| L. Harris, P F. ... | 2 | 0 |
| Pelina Clarke, P.F. ... | 2 | 0 |
| Elsie Schafer, P.F. ... | 2 | 0 |
| Helena Scott, P.F. ... | 2 | 0 |
| Ann Garratt, P.F. ... | 2 | 0 |
| Annie Rayner, P.F. ... | 2 | 0 |
| Nurse Crawshaw ... | 2 | 0 |
| E. V. Kelsey, P.F. ... | 2 | 0 |
| A. Hayward, P.F. ... | 2 | 0 |
| Elizabeth A. Ainsley, P.F. ... ... ... | 2 | 0 |
| Miss Killick, P F. ... | 2 | 0 |
| E. M. Pearce, P.F. ... | 2 | 0 |
| Nurse McDonald, P.F. | 2 | 0 |
| Nurse Hughes, P.F. ... | 2 | 0 |
| M. T. Chandlers, P.F. | 2 | 0 |
| C. Barwell, P.F. ... | 2 | 0 |
| Alice Proudfoot, P.F. | 2 | 0 |
| Nurse Lawton, P.F. ... | 2 | 0 |

### Everybody's Opinion.

[Correspondence on all subjects is invited, but we cannot in any way be responsible for the opinions expressed by our correspondents. No communication can be entertained if the name and address of the correspondent is not given, as a guarantee of good faith but not necessarily for publication, or unless one side of the paper only is written on.]

### INDOOR UNIFORM AT PUBLIC ENTERTAINMENTS.

"W. H. A." writes: It is difficult for an onlooker to understand the contention that a nurse should not go here or there in uniform. Why should a nurse be ashamed to go in uniform to any assembly that is frequented by English gentlewomen? A nurse's uniform is the badge of her profession, as is the uniform of an officer in Her Majesty's service, and one never hears it argued that an officer should not be seen at this or that entertainment in uniform. The nurses of the Seamen's Hospital attend in uniform the dances given by the naval officers at Greenwich, and for some years the committee of the Seamen's Hospital has given a dance to the nurses in the hospital. The Army Nursing Sisters are seen at all military entertainments in uniform, and in your last issue you note that Miss Norman, of Eastbourne, has given a dance at which all her nurses were present in uniform. I doubt much if evil can come of such procedure as this. What is this dread of being seen at ordinary functions in uniform? Is there one possible argument why uniform should not be worn on all occasions?

### A SPECIAL CORPS OF TRAINED NURSES FOR THE WAR.

"Sympathiser" writes: Is it not necessary that a special "corps" of trained nurses should be organised to go to Africa to nurse the cases of enteric? The whole army in Africa has 56 nurses only. The "orderlies" are without number. I know they are a fine body of men, and do most excellent work; indeed, they cannot be done without in army work. But with enteric it is different. Typhoid requires the best skilled nursing that can be obtained, and our "orderlies" have not been trained to that. I have had considerable experience in the nursing of typhoid in South America, where the disease was epidemic. Hundreds of young Englishmen annually died from it till the British Hospital took in and nursed all who applied for admission. Our young Englishmen were sent in by train, bullock cart, or even carried on shoulders of natives. Hundreds of miles from the interior our fame had gone abroad over the land. We had male nurses in our hospital, but they never nursed the typhoids; that was left to trained English women. Men cannot do it. They have not had the necessary training. It is no use applying to the War Office. "The laws of the Army Medical Department are as unalterable as the laws of the Medes and Persians" (so I was once told by a surgeon-general); but I hope I may be pardoned for suggesting that something might be done to provide skilled nursing on a big scale to look after the cases of enteric, dysentery, and pneumonia, more and more cases of which will constantly arise and undoubtedly carry off as many of our brave soldiers as the shot and steel. One person can do so little. A nurse herself has so little influence. The matrons of our big hospitals and the sisters have no time to think out these matters. The public are ignorant—very ignorant—about nursing matters generally; but they are only too willing to help if they only knew how. My heart is bleeding for the wives, mothers, sisters of England, and we ought to do all that is possible to send them back their men.

### PRIVATE NURSING.

"Nutrix" writes: I have been deeply interested in the correspondence which has appeared in your pages as to why nurses do not join institutions, and should have given my views on the subject but for the fact that my hands have been so full as to preclude me from doing so. I think one reason why many nurses who join institutions leave at the expiration of their first term is because of the favouritism which is far too prevalent. I know of an instance where the superintendent systematically favours one particular nurse; so much so that the best cases are specially selected for her, and if by chance she should be sent to a case where she is the least uncomfortable, she has only to make the fact known and another less fortunate nurse, who may perhaps have only just returned from a very trying case, is at once ordered off to replace the favourite. This I consider to be very unfair. Again, in the matter of food the same rule is observed; while the other nurses must abide strictly by the usual routine allowance the favourite can have almost anything she fancies. In the institution to which I allude this practice is so common that new nurses, who have only been attached to the home a few days, have observed and commented upon it. Perhaps some will say, Why do you not speak about it? It is no easy matter for one or two to do so. The only remedy for this kind of grievance, as it seems to me, would be in united action; and as newcomers naturally feel reluctant to take any part in a movement which might possibly damage their prospects, it is a difficult matter to secure such effective union. Our other superintendent was always a most strict disciplinarian, but at the same time she was kind to all, and was most careful to see that every nurse received the same treatment, and as a result there was no friction and there were no undercurrents of dissatisfaction. Let us have a fair field and no favour, and then I feel sure we shall see and hear less of nurses leaving institutions and setting up for themselves than we do at present.

### THE BATHING OF MALE PAUPERS, AND OTHER QUESTIONS.

"A Gentlewoman" writes: The letter of "Inquirer" on the above subject is a little difficult to follow, but apparently the writer is under the impression that "to the pure all things are no longer pure," and the advent of that gentlewomen into the nursing profession is responsible for the change. To imagine that because a woman, gentle or simple, becomes a nurse, she can set the laws of decency at defiance, and retain her own and her patients' and fellow-workers' respect, is a grave mistake. If she has lost all sense of modesty, she has no right to imagine her patients to be equally unfortunate. I have had much experience in nursing male patients, both in my training school (one of the largest hospitals in England) and since, as sister of a male flat at another important hospital. I have almost invariably found the patients self-respecting and chivalrous to the nurses, and I am a great believer in the saying that "in treating a man as a gentleman you go half way towards making him one." I never heard of a nurse being asked or permitted to pass a catheter for an adult male or to take him to the bath, and I should consider such a proposal an outrage both to the nurse's modesty and the patient's self-respect. That there are services which should be rendered to men by those of their own sex no modest woman, one would think, could deny; at the same time no true nurse would dispute that circumstances may arise in which necessity which knows no laws must be the first consideration. To unnecessarily perform these offices where male help can and ought to be forthcoming is to my mind wilfully to injure one's own and one's patient's self-respect. On the other hand, to refuse one's aid where efficient male assistance cannot be had, and one's refusal would cause pain or serious discomfort to one's patient, or perhaps danger to life or health, is both prudish, cruel, and indefensible. To the latter class of cases belongs "Inquirer's" instance of the nurse's use of the catheter when private nursing, where medical help is not available. And it is to these cases of necessity that "To the pure all things are pure" truly applies. If the ideas "Inquirer" deplores, and which she imagines only to have entered the nursing profession with the "gentlewomen" are those of modesty, she will find few to echo her complaint. But she is wrong if she thinks the "women" (of whom she is the self-constituted champion) who preceded and are working side by side with the gentlewomen are not just as modest and womanly as their sisters who have the advantage of them in social position. The "aim of a nurse's life," as your correspondent rightly says, should be "to do her best, without regard for her own wants, likes or dislikes, her one object to relieve and help those who are sick and suffering." This is the ideal of every true nurse, whatever her social standing, but it is not to be gained by ignoring and riding rough-shod over those attributes of modesty and self-respect which distinguish man from the beasts.

### The Nurses' Bookshelf.

[We invite Correspondence, Criticism, Enquiries, and Notes on Books likely to interest Women and Nurses. Address, Editor, The Hospital (Nurses' Book World), 28 & 29, Southampton Street, Strand, London, W.C.]

Home Nursing. By Sister Grace. Edited by "Isobel," of *Home Notes*. (C. Arthur Pearson, Ltd., Henrietta Street, W.C.)

This is one of a series of useful hand-books, which will be all the more appreciated because of its simplicity. Indeed, the author, Sister Grace, whose practical experience is a guarantee of knowledge, and whose style is as direct and admirable as her suggestions are valuable, confesses that her great object aimed at is simplicity, and she truly points out that the usual fault of many works of the kind is that they are too far advanced for those whom they are meant to assist. Sister Grace, avoiding such an obvious mistake, is careful to give her instructions in language which she who runs may read. In her little volume the home nurse will really find all she needs to know, and when she has mastered it she will not, if she be a sensible woman, imagine that she is qualified to undertake duties which belong to, and can only be discharged effectively by, her trained sister.

The Nurse's Pocket Diary. 1900. Price 1s. (Scientific Press, Ltd., 28 and 29, Southampton Street, Strand.)

The Scientific Press (Ltd.) can be congratulated upon the handy little diary they have just issued for the year 1900. It is small enough to carry in the pocket, and contains ample space for notes and memoranda. In an appendix there is much useful information relating to postal regulations, weights and measures, and some invaluable remarks on poisons and their antidotes. It is likewise issued in a leather case uniform with the "Nurse's Dictionary" (Miss Honnor Morten) and the "Pocket Case Book," the price of the three complete in the case being 5s., or, if in leather, 7s. 6d. We cannot imagine anything more useful or attractive as a Christmas present for a nurse than this fascinating little series, the red leather case imparting an additional charm for such a purpose. The "Pocket Case Book" is especially designed for district and private nurses, and is excellently arranged; the "Nurse's Dictionary" is too well known to require further comment, and its popularity only increases with time. We feel sure that whether taken separately, or together in a case as just issued by the Scientific Press, these little volumes will always enjoy a wide circulation among nurses.

Comprehensive Guide to Printing and Publishing. (W. H. and L. Collingridge.)

This very useful little book should be in the hands of all who are launching on the troubled sea of authorship or journalism, especially of those to whom the voyage is unfamiliar. The "Pocket Printing Guide" has been issued for many years by Messrs. Collingridge, while the "Comprehensive Guide" now before us, having already passed through ten editions, has been thoroughly revised and furnished with reliable information. The needs of business men, secretaries of societies, the clergy, and last, but not least, amateur authors, with regard to printing are duly expatiated upon and catered for, while specimens of the various kinds of type are shown and described. Many useful hints may be gleaned, especially by the uninitiated, from the remarks on methods of publishing, literary assistance, copyright, and estimates; and some space is given to the methods employed for the production of illustrations, which have recently undergone an almost complete change. The inexperienced journalist and author can learn all that he is likely to want to know on the subject of correcting proofs—a very necessary acquisition of knowledge, for, as the compiler of the "Guide" truly remarks, "the omission of an initial or a final letter, the substitution of one vowel for another, or of one consonant for another, may render what you intended to be a brilliant passage sheer nonsense, or—worse still to a correct writer—a violation of the first principles of syntactical propriety."

A Guide to Urine Testing (for Nurses and Others). Mark Robinson, L.R.C.P., L.R.C.S.Ed. (Bristol: Wright and Co. 1899. Duodec. 40 pp. Price 1s.)

The excuse offered by the young woman in "Midshipman Easy" for her undesired offspring was that "it was a very little one." The excuse will apply also in the case of this booklet. It is adapted for carrying in the pocket of a nurse's apron, and is very cheap, well printed, and strong in paper, no unimportant detail in view of the usage it is likely to receive. The pamphlet contains all that a well-trained nurse need absolutely know about testing urine. But the information afforded is very bare, and any nurse anxious to possess a good handbook would do better to provide herself with one of the small manuals (such as Wickham Legge's), from which the matter in Mr. Robinson's guide has avowedly been derived. The author has carried a striving after simplicity and brevity to a pitch which sometimes impairs the meaning of his writing; while the tables at the end might easily mislead, as no stress is laid on the fact that these tables are illustrative of extreme cases; a note should be added on the difficulties produced by such combinations as albumen and glucose, or urates and albumen, and the variations in specific gravity, consistence and colour of urine which the co-existence of these excretions constantly afford.

### Appointments.

Royal United Hospital, Bath.—Miss S. Emily Polden has been appointed Matron. She was trained at St. Bartholomew's Hospital, London. Subsequently she has been night sister, and from March, 1898, assistant matron, at Poplar and Stepney Sick Asylum.

Stanley Hospital, Liverpool.—Miss Mary Lawson has been appointed Lady Superintendent. She was trained in the Edinburgh Royal Infirmary, and since her training has been assistant nurse for one year, charge of theatre, and head nurse (or sister) for six years in the same institution.

Skipton Hospital.—Miss Alice Riley has been appointed Matron. She was trained at the London Hospital, and has since been sister at the Royal Berks Hospital for two years, and head district nurse at Skipton for two and a-half years.

### Minor Appointments.

Bristol Eye Hospital.—Miss Amy Gough has been appointed Sister-in-Charge of male and female wards. She was trained at the Bristol Royal Infirmary, and has since been sister at the Park Hospital, London, and sister at the General Hospital, Birmingham.

### To Nurses.

**In order to increase and vary the interest in the *Mirror*, we invite contributions from any of our readers in the form of either an article, a paragraph, or information, and will pay a minimum of 5s. for each contribution. All rejected manuscripts are returned in due course, and all payments for manuscripts used are made at the beginning of each quarter, i.e., January 1st, April 1st, July 1st, and October 1st.**

### Where to Go.

Hospital for Consumption, Brompton. — Christmas entertainment, December 28th, at six p.m.

### OUR CONVALESCENT FUND.

We have much pleasure in acknowledging Alison's kind contribution of 10s.

### For Reading to the Sick.

### CHRISTMASTIDE.

AND the Word was made flesh and dwelt among us (and we beheld His glory, the glory as of the only begotten of the Father) full of grace and truth.—*St. John* i.

O! blessed day, which giv'st the eternal lie

To self, and sense, and all the brute within;

Oh! come to us amid this war of life;

To hall and hovel come! to all who toil

In senate, shop, or study! and to those

Ill-warned and sorely-tempted—

Come to them, blest and blessing, Christmas Day!

Tell them once more the tale of Bethlehem,

The kneeling shepherds, and the Babe Divine;

And keep them men indeed, fair Christmas Day!

—*Kingsley.*

**Reading.**

By the Incarnation God came into the world bearing in His Person the two great necessities for man's restoration to fellowship with God—grace and truth. "The Word became flesh, and dwelt among us, full of grace and truth." "Grace and truth came by Jesus Christ." That these two great necessities might be preserved and continued in the world, He organised His Church to be the divine society in which He enshrined the treasures of grace and truth for the benefit of mankind.—*Vernon Staley.*

In Jesus Christ, then, we have the guarantee or bond of religion; He is the means of an actual communication between the soul of man and the Eternal God. "There is one Mediator between God and men, the man Christ Jesus." He is the Mediator in virtue of the very terms of His being; His office of mediation is based upon the two natures which are united in His single Person. On the one hand, as the Eternal Son, He is one with the All-Holy and Infinite God; on the other, as the child of Mary, He shares all the finiteness and weakness of our manhood; He shares with it everything except its sin. Thus He impersonates and maintains, by the very fact of being what He is, a true vital bond between earth and heaven.—*Liddon.*

Christmas hath a darkness,
Brighter than a blazing noon;
Christmas hath a chillness,
Warmer than the heat of June;
Christmas hath a beauty,
Lovelier than the world can show;
For Christmas bringeth Jesus,
Brought to us so low.

Earth strike up your music,
Birds that sing and bells that ring;
Heaven hath answering music,
For all angels soon sing;
Earth put on your whitest
Bridal robe of spotless snow;
For Christmas bringeth Jesus,
Brought for us so low. —*Christina Rossetti.*

Let not the hearts, whose sorrow cannot call
This Christmas merry, slight the festival;
Let us be merry that may merry be,
But let us not forget that many mourn;
The smiling Baby came to give us glee
But for the weepers was the Saviour born.

—*H. Coleridge.*

### Notes and Queries.

**The contents of the Editor's Letter-box have now reached such unwieldy proportions that it has become necessary to establish a hard and fast rule regarding Answers to Correspondents. In future, all questions requiring replies will continue to be answered in this column without any fee. If an answer is required by letter, a fee of half-a-crown must be enclosed with the note containing the enquiry. We are always pleased to help our numerous correspondents to the fullest extent, and we can trust them to sympathise in the overwhelming amount of writing which makes the new rules a necessity.**

**Every communication must be accompanied by the writer's name and address, otherwise it will receive no attention.**

*Exchange.*

(127) Has any nurse a "Barnes" she would sell cheap or exchange for "Gubb's Midwifery" (new).—*Nurse E.*

Had "Nurse E." given us permission to print her name and address her request would have been put in "Wants and Workers," and so saved much forwarding of replies,

*Bandage and Toenails.*

(128) Will you kindly inform me if there is any bandage or appliance made which a boy could wear who has had his shoulder dislocated playing football so that he could continue playing in the next football season with less risk? I have also had several boys with ingrowing toenails. Is this entirely due to tight or pointed toes in boots and shoes? What is the best preventative, and also the most effectual remedy?—*Nurse-Matron.*

These are matters in regard to which you should consult your medical officer. Speaking generally, and with no reference to the individual case, it may be remarked that no appliance is likely by main force to hold a shoulder joint together which is liable to dislocation. What an appliance might do would be to restrain the arm from making those movements which would be likely to lead to a recurrence of the accident. Whether the captain of a team would care to have a boy whose movements were so restricted is another question, and it must be remembered that boys gain little from games in the playing of which they are debarred from making efforts to excel. In regard to ingrowing toenails. This is largely due to boots with pointed toes, but it must not be imagined that the cure is to wear large and clumsy boots. What is wanted is a shoe with sufficient room about the toes to enable them to move freely, and so well fitting that when laced up it shall be impossible for the toes to be jammed together by the foot sliding forward within the shoe. The lacing is the essential thing. Even in a badly-shaped boot the toes may often be prevented from crowding together if the boot is well laced. If the shoes are merely made loose the boy will develop corns as well, without in any way relieving his toe nails. As a means of prevention it must be remembered that frequent washing of the feet is the most important thing, and if there is any tendency for the feet to become offensive bathing them with a lotion of (1 to 40) carbolic acid.

*Gargle.*

(129) Will you kindly tell me how you would make a good gargle for the throat, from honey and vinegar (if these would make a good gargle); also (2) how you would make a good substitute for a mustard plaster (that is in an urgent case) when you are too far from town to procure one. What would be the simplest and most effectual way?—*An Interested Reader of "The Hospital."*

Honey freshly mixed, either with vinegar or lemon juice in equal proportions, is nice, not as a gargle, but to moisten an inflamed mouth and throat with, and to assuage thirst and coughing. 2. Mustard is to be obtained in every household or village shop, and anyone can make a plaster at a moment's notice by mixing it with a little water and spreading it on a sheet of paper.

*Aged Seventy.*

(130) Will anyone kindly tell a nurse if there is anything to be worn or done to prevent the bowel from coming down the rectum for an old lady of 70?

Prolapse of the rectum is a fairly common disease among elderly people, arising sometimes from piles, sometimes from polypus, and often from mere relaxation of the parts. It can in many cases be relieved by medical or surgical treatment. Evidently a medical practitioner should be consulted.

*Au Pair.*

(131) A private nurse would be glad to know if there are any hospitals in London where she could go between her cases by giving her services and paying a small sum weekly?

Such an arrangement as you suggest is unpractical.

*In the Van.*

(132) Will you kindly tell me of the best book that is published that will enable a private nurse to keep herself in touch with treatment of diseases, &c., many of which she does not come across in private work, and after having left hospital for some years one gets, so to speak, so very much behind the times.—*Private Nurse.*

The Post Graduate Nursing Lectures in THE HOSPITAL "Mirror" are designed entirely to meet this want. Dr. Watson's recently published "Handbook for Nurses" (5s., Scientific Press) is highly spoken of, and should prove useful to trained nurses who wish to keep themselves in the van of progress.

*Abroad.*

(133) Will you kindly tell me the addresses of any private nursing institutions in Rome, Florence, or elsewhere on the Continent? Is the Hollond Institute still in existence?—*Nurse.*

The address of the Hollond Institute is 1, Tavistock Chambers, Bloomsbury, W.C. We do not care to mention private institutions, but the English Consul or chaplain at either Rome or Florence would possibly give you the required information.

*A Correction.*

(124) The Nurses' Hostel, Francis Street, W.C., writes the managing director, offers facilities for a nurse to quarantine after disinfecting, but is not in a position to disinfect a nurse after nursing an infectious case. The disinfecting must be done before leaving the patient's house.

### Travel Notes.

By Our Travelling Correspondent.

### XLII.—WINTER RESORTS—*(continued).*

### Meran in Austria.

This favoured spot is a health resort all the year round; in winter, its wonderfully equable climate and brilliant sunny sky is specially suited to those in the early stages of pulmonary complaints; in spring it offers the whey cure, and in autumn the grape cure. The journey occupies forty hours, if taken without a break, but no one would travel thus unless imperatively compelled. Good stopping places are, first, Laon or Rheims, both interesting places, Bâle, and Innsbruck. This last is a charming spot, and well worthy of a residence en route to Meran. As to cost, first class to Meran is £7 13s., and second £5 6s. 6d., and you may occupy 15 days in reaching your destination, so that you can well spend ten days at fascinating Innsbruck, if so inclined. The coinage of Austria is not to our advantage like that of France, slightly, or that of Italy considerably. A florin, still the most ordinary coin in Austria, is about the worth of one shilling and ninepence. For an English sovereign the average rate of exchange is twelve florins, roughly speaking, nineteen shillings. Meran is not a cheap place, but there are various pensions, and for a long stay favourable terms could probably be made. For those unable to walk much the Gisela Promenade is always a pleasant resort. Close by is the Corhaus, closed in summer; here a good band plays daily. Further on is the Stefanie Promenade, and various other nice places easy of access. Of longer excursions there is no end; that to Schloss Tirol, occupying three or four hours, is very interesting—the road is delightful, and there is a magnificent view from the Castle itself. The afternoon is the best time for effect. Five miles to the south of Meran is the Château of Lebenberg, now used as a pension; terms moderate. I am told this is a very comfortable house, but I do not know it myself. It is a grand old building, and quite worth a visit. There is so much to see in this lovely neighbourhood that you would not exhaust its beauties in many months.

### Pau.

This is a delightful place of residence for the winter, in some respects preferable to the French or Italian Riviera; it is not so warm nor quite so sunny, perhaps, but then there is no mistral, an advantage not to be overlooked. Its climate is very equable, and has strongly sedative effects. I know it at all seasons, and even in the height of summer, when fashion flies to the mountains and the humbler inhabitants go about contentedly in the sparsest raiment, it is still delightful. The mornings and evenings are fresh and lovely, and after all, in really hot weather, none but lunatics desire to make strenuous exertion in the middle of the day. The cost of the journey is £6 10s. 3d. first-class, and £4 9s. second, viâ Dover and Calais; by Newhaven and Dieppe, £5 7s. 11d. first, and £3 15s. 11d second. The best stopping place is, of course, Paris, and if a second halt is needed I think I should make it at Tours. If you go straight through without a break the time occupied on the road is close on 24 hours, viâ Dover, and an hour more by Dieppe. There are plenty of good hotels, and for a lengthened stay, if you are not particular as to your rooms looking to the south, you can arrange for pension terms at eight francs per day, but the moment that aspect is of importance the terms run up surprisingly. There are no really cheap hotels at Pau; it is too entirely a *ville de luxe*. There are one or two pensions, but I hardly think they would be found to be much cheaper. As to residence in rooms of your own, the advantage would be discounted by the high price of provisions; in all places catering so much for English and Americans living is dear. The attractions of the town are considerable. The Place Royale, looking to the magnificent range of the Pyrenees, is the lounge *par excellence* of rank and fashion; this splendid promenade now extends from the Royal Park on one side to the Parc Beaumont on the other, and has no rival in Europe in its own particular line of beauty. The band plays here (Pau has a large garrison) twice a week, and on the parade ground other days. There are excellent concerts in the Casino and a good theatre. The golf club (subscription £3) is a great addition to the pleasures of Pau; the links are very near, at Bilhère, a pretty little village, where the great Henri Quatre was foster nursed. Here, too, is a good tennis club, and there is a subscription pack of hounds, so altogether the men are as well catered for as the women in this gay little city. It is a capital centre for mountain excursions, and the drives and walks are numerous and beautiful.

### Grasse.

This lovely town is associated in our minds with scent of all kinds. The flowers used in the distilleries are grown on vast terraces forming nursery gardens. The whole process, from the growing of the flowers to the bottling, sealing, and packing the perfumes, is most interesting. Grasse is 13 miles north of Cannes, and from its greater distance from the sea is favourable for cases requiring a sedative climate. The journey viâ Dover is £7 12s. 6d. first class, and £5 4s. 1d. second; by Newhaven and Dieppe, £6 10s. 10d. first class and £4 10s. 8d. second. Stopping places the same as for Nice or Mentone. There is at present a scarcity of hotel accommodation. One, the Grand Hotel, is rather expensive; where there is but little competition this is usually the case. The other, Hotel Muraour, much more moderate and very comfortable, is in the centre of the town, and, therefore, not quite so suitable for invalids. The surrounding country is lovely, with plenty of excursions suited for invalids or the able-bodied. The town itself is one of the most picturesque in that country, which seems to have been constructed and built upon to delight the eye.

---

### TRAVEL NOTES AND QUERIES.

Hyeres (Arethusa).—Hyères is cheaper than the majority of places on the French Riviera, and it has the advantage of being very near to Marseilles. It is, however, much tormented by the mistral. I fancy St. Raphael might suit you better. Hyères is some distance from the sea, but it has great advantages—a capital centre for excursions. St. Raphael is very quiet as yet, though it is developing rapidly. Better than either of these places is Bordighera, if your doctor approves. You must, however, expect higher prices than usual on the Italian Riviera on account of Her Majesty's visit.

Perugia (Artist).—It is possible to live there very cheaply, but not for ladies alone. If, as you hope, your brother goes with you, you can take two or three rooms, live very cheaply, and sketch every day without exhausting the artistic bits. The interior of the cathedral is disappointing, but I think you could obtain permission to paint inside; the outside, however, with its ideal surroundings, is much more enthralling. (2) I should put it at about 30 lire. Living is cheap in Italy, and an English sovereign is worth from 27 to 28 lire.

German in Konigswinter (Bee).—You can get on quite well on the Rhine without German. On all the beaten tracks the waiters speak English. The only place you may find a little difficulty is on the railways, but everyone is very kind and helpful. A slight knowledge of French is often helpful, but really you will not want it on the Rhine. I have never been there earlier than May, and I think before that it would be very raw and cold. I think the ideal month would be June, and failing that September. At St. Servan or Paramé (they are close together) living is cheap, and may be had for 5s. a day. I will give you addresses if you write to me again. The journey also cheap and easy—first-class return, £2 12s.; second return, £1 19s. Second-class is very comfortable, and even has an advantage—it is not so crowded. With regard to the climate it is mild, but not warm. The rainfall is not so great as in England, and there is more sun. If you are only a little delicate, and have no decided lung trouble, I think you might find a stay there beneficial. I have myself spent two winters at Dinard, across the Bay. I spoke at some length in Article XXI. of the Bay of St. Malo, which, if you look it up, will give you some useful information. The following one (XXII.) deals with Le Mont St. Michel, which would form one of your first excursions. Thank you for the kind things you say of "Travel Notes." I am glad they are useful and give pleasure.

